# Observation of Both Skilled and Erroneous Object Lifting Can Improve Predictive Force Scaling in the Observer

**DOI:** 10.3389/fnhum.2019.00373

**Published:** 2019-10-22

**Authors:** Guy Rens, Marco Davare

**Affiliations:** ^1^Movement Control and Neuroplasticity Research Group, Department of Movement Sciences, Biomedical Sciences Group, KU Leuven, Leuven, Belgium; ^2^Leuven Brain Institute (LBI), KU Leuven, Leuven, Belgium; ^3^Department of Clinical Sciences, College of Health and Life Sciences, Brunel University London, Uxbridge, United Kingdom

**Keywords:** action observation, hand movement, motor planning, error prediction, skilled action, sensorimotor

## Abstract

Recent studies have highlighted that the observation of hand-object interactions can influence perceptual weight judgments made by an observer. Moreover, observing salient motor errors during object lifting allows individuals to update their internal sensorimotor representation about object weight. Embodying observed visuomotor cues for the planning of a motor command further enables individuals to accurately scale their fingertip forces when subsequently lifting the same object. However, it is still unknown whether the observation of a skilled lift is equally able to mediate predictive motor control in the observer. Here, we tested this hypothesis by asking participants to grasp and lift a manipulandum after observing an actor’s lift. The object weight changed unpredictably (light or heavy) every fourth to sixth trial performed by the actor. Participants were informed that they would always lift the same weight as the actor and that, based on the experimental condition, they would have to observe skilled or erroneously performed lifts. Our results revealed that the observation of both skilled and erroneously performed lifts allows participants to update their internal sensorimotor object representation, in turn enabling them to predict force scaling accurately. These findings suggest that the observation of salient motor errors, as well as subtle features of skilled motor performance, are embodied in the observer’s motor repertoire and can drive changes in predictive motor control.

## Introduction

Skilled hand movements are essential throughout our daily life. It has been well established that dexterous object manipulation not only relies on tactile feedback but also on anticipatory sensorimotor mechanisms. Performing hand-object interactions allows internal object representations to be formed. In turn, these internal sensorimotor representations can be retrieved to enable anticipatory planning of digit forces for future object manipulations (e.g., see Johansson and Westling, 1988). It has been argued that predictive force scaling requires an association between intrinsic object properties, for example, size or texture, and the object weight, which are experienced by visual and tactile feedback, respectively (Baugh et al., 2012). In addition, other research groups have demonstrated that object weight is not only perceived via somatosensory inputs but can also be retrieved through vision, and that visual weight judgments are associated with the actual object weight (Runeson and Frykholm, 1981; Bingham, 1987). Finally, it has been established that the object lifting phase conveys critical information for mediating weight judgments: observers mostly rely on the duration of the lifting movement for generating weight perception (Shim and Carlton, 1997; Hamilton et al., 2007).

The influence of action observation on both weight perception and lift performance was first investigated by Meulenbroek et al. (2007): they demonstrated that when both the actor and participant had an incorrect weight prediction, lifting performance errors made by the participant are reduced, but not eradicated, after observing the actor making typical lift errors. In addition, it was shown in a more recent study by Uçar and Wenderoth (2012) that observation of different types of hand movements can alter grip force generation during object grasping: prior to grasping an object, participants were asked to observe an actor either touching or squeezing an object. The latter condition led participants to produce larger grip forces. Finally, it has been demonstrated that when individuals observe lifting errors, they are able to differentiate object weight based on kinematic cues and, in turn, scale their fingertip forces more accurately in upcoming trials (Reichelt et al., 2013). Although these studies have shed light on how action observation can mediate anticipatory motor control in the observer, they only focused on the observation of explicit hand-object interactions [different movements (Uçar and Wenderoth, 2012) or salient movement errors (Meulenbroek et al., 2007; Reichelt et al., 2013)] and not on more subtle kinematic features of skilled motor performance.

To the best of our knowledge, only few studies have compared how observing erroneous and skilled object interactions can mediate predictive force scaling. For example, using the size-weight illusion, Buckingham et al. (2014) highlighted that predictive force scaling in the observer is significantly better after observing erroneous compared to skilled lifting. That is, when participants had to lift a large, but unexpectedly light object for the first time, those who observed typical overestimation errors on the same object would predict the actual weight more accurately. Interestingly, when investigating how corticospinal excitability (CSE), probed with transcranial magnetic stimulation (TMS), was modulated during lift observation, Buckingham et al. (2014) found that only during the observation of skilled lifts, CSE was modulated by object size: CSE modulation was significantly higher in response to the observation of a skilled lift of the larger object compared to the smaller one. However, during observation of erroneous lifts on the same objects, the effect of object size on CSE modulation was eradicated. As such, it seems that, when observing skilled object lifting, object size is the critical factor for extracting object weight and driving CSE changes; while when observing erroneous lifts, kinematic cues, not size, have a predominant effect. Arguably, it seems plausible that when a lifting error is observed by participants, the unexpected object kinematics drive the observer to shift their attention toward the object kinematics and not size, improving the observer’s predictive force scaling and altering the underlying CSE modulation.

With specific interest to TMS studies, it has been demonstrated that CSE is similarly modulated during action execution and observation (Fadiga et al., 1995). Because of this, observation-driven modulation of CSE has been coined as “motor resonance” and has been argued to be driven by the putative human mirror neuron system (hMNS) (Rizzolatti et al., 2014). Interestingly, motor resonance has been shown to be modulated by parameters indicating object weight such as object size (Alaerts et al., 2010b; Buckingham et al., 2014), movement kinematics (Alaerts et al., 2010b) and muscle contraction (Alaerts et al., 2010a). Thus, given that the putative hMNS can mediate motor resonance to object weight during lifting observation, it is hypothesized that this induced motor resonance could be used to extract object weight information from observed actions and could mediate predictive force scaling in the observer.

In the current study, we aimed to specifically investigate whether observation of skilled object lifting can drive changes in internal sensorimotor representations when a similar action observation strategy is used for both erroneous and skilled lifts. For this, we asked participants to perform an object lifting task in turns with an actor. The weight changed every fourth to sixth trial of the actor’s lifts so that participants could not predict this change. However, the weight lifted by the participants always matched the weight of the actor’s preceding trial. As such, participants could monitor during action observation whether the object weight was changed (or not) and then use this information for their forthcoming trial. To investigate whether observation of skilled and erroneous lifts mediate predictive force scaling differently, we controlled three factors: (1) we used objects that are identical in appearance to exclude that size and other visual cues could be used to predict object weight. (2) Similarly to the study of Reichelt et al. (2013), participants were familiarized to the experimental protocol and object weights. (3) In contrast to the study of Reichelt et al. (2013), participants were informed that they would have to focus on the observation of either skilled or erroneous object lifting. We argue that these factors would allow participants to better understand the task goal and would lead them to actively search for cues that could indicate object weight (such as movement kinematics or hand contraction) during observation of both types of lift performance. For erroneous lift observation, we expected, as been demonstrated by Reichelt et al. (2013), that participants would be able to predictively scale their fingertip forces more accurately after observing an erroneous lift compared to performing the task alone (i.e., no observation). In addition, we expected that predictive force scaling could also be improved after observing skilled lift performance considering that motor resonance is modulated by parameters, such as movement kinematics and hand contraction states, which reflect object weight (Alaerts et al., 2010a). Accordingly, motor resonance might enable observers to update their sensorimotor memory. For example, when observing an actor skilfully lifting a heavy object, in a context in which the observer expected the object to be light, motor resonance should reveal the actual object weight through the observed lifting parameters (e.g., observing a larger muscle contraction than expected). When these observed parameters are integrated into the motor command to be planned by the observer (i.e., muscle contraction for a light object), the mismatch should indicate that the object is heavier than expected. Consequently, the observer can update his motor command by relying on object-related information driven by motor resonance.

In conclusion, we hypothesized that observing skilled or erroneous object lifting could mediate predictive force scaling similarly. We expected that when participants observed an actor’s lift, irrespective of lift performance type, they would perceive the weight change and update their sensorimotor memory by relying on information about object weight mediated by motor resonance. Accordingly, we expected that participants would not produce a lifting error after observing either lift performance type.

## Materials and Methods

### Participants

A total of 14 participants (6 males and 8 females; mean age = 19.7 ± 2.9 years) were recruited from the student body of KU Leuven to participate in the current study. All participants were right-handed (self-reported), had normal or corrected-to-normal vision, were free of neurological disorders and had no motor impairments of the right upper limb. The study was conducted in accordance with the declaration of Helsinki and was approved by the local Ethical Committee of the Faculty of Biomedical Sciences, KU Leuven (s60072). Participants were financially compensated for their participation. Data of one participant were rejected after the data analysis stage due to high inconsistencies in grasping patterns throughout the experiment.

### General Procedure

**FIGURE 1:**
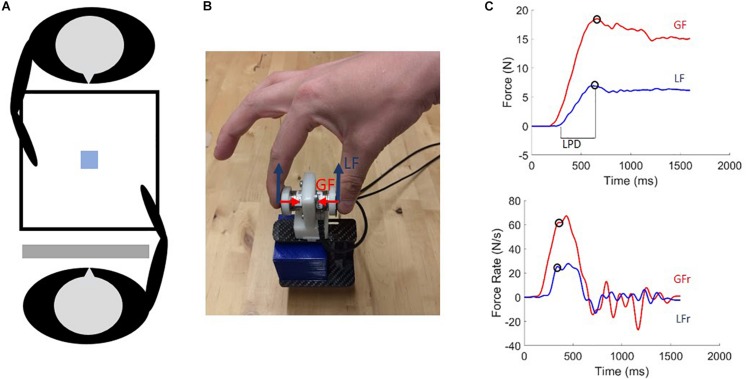
**(A)** Experimental set-up: The participant and actor are seated opposite each other in front of a table on which the manipulandum was positioned and a switchable screen was placed in front of the participant’s face. **(B)** Photo of the grip-lift manipulandum used in the experiment. Load force (LF: blue) and grip force (GF: red) vectors are indicated. **(C)** GF and LF typical traces (upper) and their derivatives (lower) for a skilled lift. Circles denote first peak values used as parameters. Loading phase duration (LPD) is indicated on the upper panel.

Participant and actor were comfortably seated opposed to each other in front of a table (for the experimental set-up see Figure 1A). Participants were required to grasp and lift a manipulandum (see section “Data acquisition”) that was placed in front of them (1) either repeatedly (see section “SOLO Condition”) or (2) in turns with the actor (see section “Dyadic Conditions”). Participants and actor used their entire right upper limb to reach for the manipulandum and were asked to grasp it with the thumb and index finger only (precision grip). Participants and actor were required to lift the manipulandum smoothly to a height of approximately 3 cm and to keep the grasp-and-lift movement consistent throughout the entire experiment. Additionally, participants and actor were required to place their hand on a predetermined resting position on their side of the table between trials, at a distance of approximately 25 cm from the manipulandum. This was done to ensure consistent reaching movements across trials. Each trial initiated with a neutral sound cue (“start cue”), indicating that the movement could be initiated. Trials lasted 4 s to ensure that participants and actor had enough time to reach, grasp and lift the manipulandum smoothly at a natural pace. Inter-trial interval was approximately 5 s, during which the weight of the manipulandum could be changed. A transparent switchable screen (Magic Glass), placed in front of the participants’ face, became transparent at trial onset and turned back to opaque at the end of the trial. The screen remained opaque during the inter-trial interval.

### Experimental Conditions

We used an experimental set-up similar to the study of Reichelt et al. (2013). Participants always performed the solo condition first in order to familiarize themselves experiment. After this condition, participants performed two dyadic conditions, i.e., erroneous lift observation (“ERROR”) and skilled lift observation (“SKILLED”). Each dyadic condition was performed two times in a counterbalanced order within and across participants.

#### Solo Condition (“SOLO”)

Participants repeatedly lifted the manipulandum themselves, therefore performing all trials. The weight of the object changed between 1.5 N (light, “L”) and 6.2 N (heavy, “H”) after a pseudo-random amount of trials of the same weight. The number of trials per weight sequence (i.e., sequential lifts of the same weight) varied randomly between 3 and 6 trials. Thus participants could not predict when the weight change would occur based on the number of lifts. Participants completed eight transitions from each weight to the other (i.e., from 1.5 N to 6.2 N and vice versa). This provided eight trials per weight transition, which were used to familiarize participants, assess baseline sensorimotor memory effects (for example see: Johansson and Westling, 1984) and use for comparison with the dyadic conditions. Lastly, considering that Reichelt et al. (2013) used six weight transitions, we decided to include two more as, in the dyadic conditions, the actor had to execute two performance types (ERROR and SKILLED). As such, the actor might have been more prone to lifting “incorrectly” (e.g., lifting erroneously when skilfully was required or vice versa) causing us to potentially remove an entire weight sequence based on the actor’s mistake.

#### Dyadic Conditions

Between the end of the SOLO condition and the start of the first dyadic condition, participants were instructed on lifting errors i.e., incorrect scaling of fingertip forces due to wrong estimation of object weight. They were told that in the dyadic conditions they would have to lift the manipulandum in alternation with the actor and that the object weight presented in their trial would always be identical to the weight lifted by the actor in his preceding trial. It was also mentioned that the object weight would always change first for the actor and then would be the same for the participant. Finally, participants were asked to avoid making lifting errors (i.e., scale fingertip forces accurately to the object weight, as perceived during the actor’s preceding lift) and, importantly, they were told to use cues from the actor’s movement to estimate object weight. However which movement cues could be relevant or which strategy could be used were not discussed to ensure that participants would rather rely on a self-generated strategy than on the experimenter’s instructions. After receiving the task instructions, participants performed the two dyadic conditions. As in the SOLO condition, there were eight transitions from one weight to the other after a pseudo-random amount of trials. During the dyadic conditions, actor and participant alternatingly lifted the manipulandum between 3 and 6 times before the weight changed (3 and 6 included; same amount of repetitions for each person). As such, each weight sequence within the dyadic condition, consisted of 6 to 12 trials in total.

Because each dyadic condition took twice the amount of trials in comparison with the SOLO condition, both dyadic conditions were divided into two blocks with a break in between them. This was done to prevent fatigue affecting observation and movement performance. Dyadic block order was counter-balanced between participants (i.e., half started with ERROR and half with skilled) and within participants (two blocks of the same dyadic condition were never performed back to back). In addition, participants were informed prior to the start of a block which lifting performance type the actor would use. Although both dyadic conditions consisted of two separated blocks, data is presented pooled per condition. In the SKILLED condition, the actor always scaled his fingertip forces correctly to the weight that was presented to him. As a result, the participant could only extract information about object weight by observing skilled lifts. In the ERROR condition, the actor incorrectly scaled his fingertip forces when the new weight was presented. This lifting error was made only in the first trial after the weight change. In all other trials of the same weight sequence of the ERROR condition, the actor would perform a skilled lift of the manipulandum. Thus, in the ERROR condition, participants could perceive a weight change by looking for lifting errors. Importantly, the lifting error made by the actor was intentional (“artificial”) due to the experimental set-up (see section “Data Acquisition”). Lastly, one of the authors (GR) served as an actor for all experiments.

### Data Acquisition

A grip-lift manipulandum consisting of two 3D force-torque sensors (Nano17, ATI Industrial Automation, Apex, NC, United States) was attached to a custom-made carbon fiber basket in which different objects (cubes) could be placed (For an example of the manipulandum see Figure 1B). The total weight of the manipulandum was 1.2 N. The graspable surface (17 mm diameter and 45 mm apart) of the force sensors was covered with fine sandpaper (P600) to increase friction. The objects were 3D-printed cubes of 5 × 5 × 5 cm, filled with different amounts of lead particles to create weights of 0.3 N (“light”) and 5.1 N (“heavy”), therefore, the total weight were 1.5 N and 6.2 N for the light and heavy weight, respectively. To exclude all visual cues about weight, cubes were hidden under the same paper cover. It is noteworthy that cubes were changed manually between each trial (even for trials without weight change) to ensure that participants could not use sound cues to predict weight changes. Second, given the actor was responsible for changing cubes between trials, he always knew what weight would be presented in the upcoming trial. Therefore, the over- and underestimation lift errors related to object weight were made intentionally (“artificially”) by the actor and not by a wrong prediction of object weight. Custom-made scripts were compiled in MATLAB (Mathworks) for both data acquisition and processing.

### Data Analysis

Force signals were sampled in 3D at 1000 Hz and smoothed using a 4th order, zero-phase lag, low-pass Butterworth filter with a cut off frequency of 15 Hz. Grip force (GF) was defined as the exerted force (on the force sensors) perpendicular to the normal force. Load force (LF) was defined as the exerted force parallel to the normal force (Figure 1B). GF and LF were computed as the sum of the respective force components exerted on both sensors. Additionally, grip force rate (GFr) and load force rate (LFr) were calculated by computing the first derivative of GF and LF. Finally, we calculated the loading phase duration (LPD) by measuring the latency between LF onset (LF > 0.05 N) and an approximation of object lift off (LF > 0.95 ^∗^ total object weight) (Figure 1C). Peak force rate values, not peak force values, are presented in the results as it has been demonstrated that these force parameters are a reliable indicator of predictive force scaling (Johansson and Westling, 1988; Gordon et al., 1991; van Polanen and Davare, 2015). These force parameters were compared based on only the first and second trials after the weight change for both participant and actor, as it has been demonstrated that individuals adapt to the actual object weight after one trial (Gordon et al., 1993). As such, these trials allowed us to investigate (1) the baseline for over- and underestimation of object weight by participants during the SOLO condition, (2) the movement kinematics of the actor in the ERROR and SKILLED condition and (3) whether the ERROR and SKILLED conditions alter the typical over- and underestimation of object weight and could mediate accurate predictive force scaling.

#### Statistical Analysis

For statistical analysis of peak force rate values, we normalized the data of the actor and each participant, as some of them altered their general force pattern over time during the experiment, although they were informed to maintain a consistent grasping pattern. Considering that we were primarily interested in the first two trials after the weight change, we normalized the peak force rate values and LPD of the first two trials by dividing them by the respective values of the third trial performed by the same person in the same weight lifting sequence. For example: If a participant had to grasp five heavy weights repeatedly, all parameters of the first two trials were divided by the respective parameters of the third trial performed by this person in this same sequence. As such, the first two trials are expressed as a ratio to the third trial and the third trial would have a value of 1 for each parameter. If any of the measured parameters in the third trial of the weight sequence was an outlier relative to this condition (value larger or smaller than mean ± 2 SD’s), then the entire sequence of weight repetitions was discarded. We chose to compute ratios based on the third trial of a weight sequence because the weight could change every 6th to 12th trial. As such, in each sequence, participants and actor performed minimally three trials but potentially more. Using this procedure, the over- and underestimations of object weight are always expressed in relation to the force pattern of skilled lifting during that specific time point and take these potential changes over time into account.

We performed repeated-measures ANOVAs to investigate differences in the weight change trials between conditions. These ANOVAs were performed separately for the “person” (actor or participant pool), the weight change (heavy-to-light or light-to-heavy) and each parameter (LFr, GFr or LPD). For example, we performed one separate ANOVA for LPD of the light-after-heavy weight changes for the actor’s data. For the participants’ data, we used two within-factors: LIFT NUMBER (first and second trial after the weight change) and CONDITION (SOLO, ERROR, and SKILLED). For the actor’s data we used two within-factors: LIFT NUMBER (first, second and last trial after the weight change) and CONDITION (ERROR and SKILLED). For the actor, we decided to include the last trial after the weight change (which was also normalized against trial 3) to control for his consistency throughout the entire sequence. We did not include the last trial after the weight change in the analyses of the participants’ data as explained in section “Data Analysis.” Please note that due to the alternating task, the first and second actor trials refer to trials 1 and 3 after the weight change. The first and second participant trials refer to trials 1 and 2 after the weight change in the SOLO condition and to trials 2 and 4 after the weight change in the SKILLED and ERROR conditions. The last trial of the actor can refer to any uneven number between 5 and 11 (both numbers included). Lastly, in the case where the last actor trial of a sequence was also the third trial (i.e., the trial to which other ones were normalized), it was excluded for calculating the actor’s average performance. Comparisons of interest exhibiting statistically significant differences (*p* ≤ 0.05) were further analyzed using the Holm-Bonferroni test. All data presented in the text are given as mean ± standard error of the mean (SEM).

## Results

We aimed to investigate whether action observation can drive changes in internal sensorimotor representations, which would further translate into changes in predictive motor control. To address this issue, we compared three conditions. In the solo condition (“SOLO”), participants repeatedly lifted the objects for familiarization purposes and to assess baseline sensorimotor memory effects caused by an unexpected weight change. In the dyadic conditions, participants lifted series of objects in alternation with an actor. Participants were informed that they would always have to lift the same object weight as the actor. For this reason, participants could use observed kinematics to perceive object weight and consequently update their internal sensorimotor representation. In the error observation condition (“ERROR”), the actor would make an artificial lifting error when the weight would change from light to heavy (i.e., “undershoot”) or from heavy to light (i.e., “overshoot”). The actor would then correctly scale his fingertip forces in the following trials. In the skilled lift observation condition (“SKILLED”), the actor would always apply correct fingertip forces. These two action observation conditions allowed us to investigate whether individuals respond differently to error vs. skilled actions, in order to plan their own motor command following an unexpected object weight change.

### Observers’ Lifting Force Parameters: Light-After-Heavy Weight Changes

**FIGURE 2:**
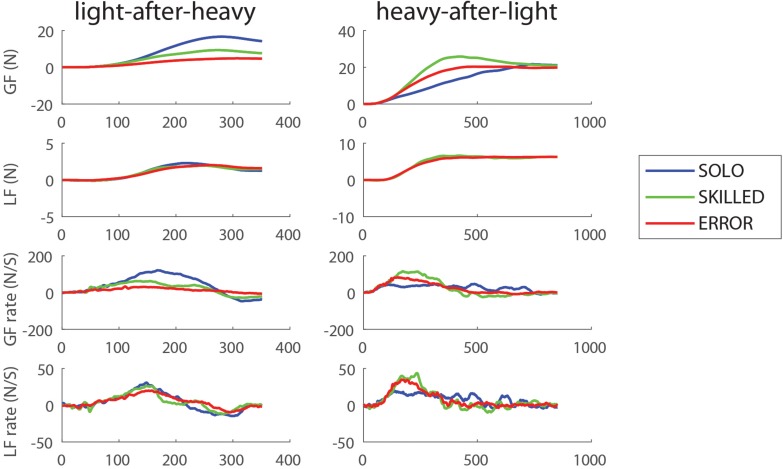
Lifting performance example for the first trial after the weight change. Typical traces showing the evolution of the different force profiles over time for one participant for the three conditions: grasping with incorrect weight expectations (SOLO), grasping after observing a skilled lift (SKILLED) and grasping after observing a lifting error (ERROR). From top to bottom: Grip force (GF), load force (LF), grip force rate (GFr) and load force rate (LFr) for light-after-heavy **(left panel)** and heavy-after-light **(right panel)**.

The left panels of Figure 2 shows the averaged force profiles of a typical participant for the first trial of each experimental condition for the light-after-heavy weight change. When a participant scales his fingertip forces in anticipation of a heavy object (although it is actually light), more force than required will be applied (Johansson and Westling, 1988). Figure 2 suggests that the participant was able to downscale his force parameters after observing an erroneous lift (ERROR; red profiles) compared to the SOLO condition (blue force profiles). In addition, Figure 2 also suggests that the participant was also able to downscale his fingertip forces after observing a skilled lift (SKILLED; green profiles). For data analysis purposes, we only included the first and second trials following a light-after-heavy weight change (second trial is not shown on Figure 2; but improvements from trials 1 and 2 for each condition are shown on Supplementary Figure S1). Values were normalized: in case of the light-after heavy weight changes, force parameters with ratios >1 indicate weight overestimation (i.e., scaling fingertip forces for heavy although the object is light). These effects are opposite for the loading phase duration (LPD): a ratio value <1 indicates a shorter LPD. All group averages of the participants for the light-after-heavy weight changes can be found in Table 1.

**TABLE 1:** Mean values for the participants’ lifting performance is presented as mean ± SEM.

		**SOLO**		**Skilled lift observation (SKILLED)**		**Erroneous lift observation (ERROR)**	
				
		**Trial 1**	**Trial 2**	**Trial 1**	**Trial 2**	**Trial 1**	**Trial 2**
Light after heavy	pLFr	1.83 ± 0.26	1.34 ± 0.11	1.43 ± 0.09	1.18 ± 0.17	1.11 ± 0.09	0.99 ± 0.99
	pGFr	2.00 ± 0.15	1.46 ± 0.10	1.69 ± 0.14	1.16 ± 0.13	1.20 ± 0.08	1.18 ± 0.07
	LPD	0.83 ± 0.05	1.04 ± 0.05	0.88 ± 0.22	1.21 ± 0.04	1.06 ± 0.06	1.13 ± 0.05
Heavy after light	pLFr	0.86 ± 0.04	1.06 ± 0.03	1.00 ± 0.04	1.24 ± 0.07	0.95 ± 0.03	1.00 ± 0.04
	pGFr	0.87 ± 0.04	1.12 ± 0.06	1.02 ± 0.06	1.22 ± 0.08	0.98 ± 0.05	1.08 ± 0.05
	LPD	1.51 ± 0.06	1.07 ± 0.03	1.27 ± 0.04	1.00 ± 0.03	1.23 ± 0.04	1.05 ± 0.02

*Values represent the normalized peak load force rate (pLFr), peak grip force rate (pGFr) and loading phase duration (LPD) for the first and second trials of the weight sequence blocks of all three conditions (SOLO: performing the lifting task alone, SKILLED: skilled lift observation, ERROR: erroneous lift observation). Normalization was done against the third trial after the weight change. Values above 1 for the peak force values indicate that participants used more force than required, which should be opposite for LPD (lower than 1). In contrast, values below 1 for the peak force values indicate that participants used less force required (above 1 for LPD). No statistics or significance shown in the table.*

#### Load Force Rates

**FIGURE 3:**
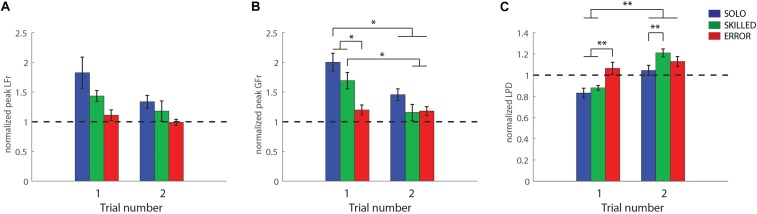
Light-after-heavy weight changes: Participant group averages for the first and second trials after the weight changed from heavy-to-light for the three conditions [grasping with incorrect weight expectations (SOLO), grasping after observing a skilled lift (SKILLED) and grasping after observing a lifting error (ERROR)]. **(A)** Peak load force rates, **(B)** peak grip force rates and **(C)** loading phase durations. All data is represented as a ratio (normalized to skilled lifting; first and second trial of the participants divided by the third trial of the same weight sequence block). A ratio >1 for peak grip force rates and peak load force rates (and a ratio <1 for loading phase durations) indicates that participants overestimated object weight. All data is presented as the pooled mean ± SEM. ^∗∗∗^*p* < 0.001, ^∗∗^*p* < 0.01, ^∗^*p* < 0.05.

Repeated-measures ANOVA for load force rates revealed that only the main effects, CONDITION (*F*_(2,24)_ = 5.07, *p* = 0.01, η^2^*_*p*_* = 0.30) and REPETITION (*F*_(1,12)_ = 9.58, *p* = 0.01, η^2^*_*p*_* = 0.44) were significant (but not the double interaction effect). The *post hoc* analysis of REPETITION indicated that participants scaled their load forces (peak LFr), significantly better in the second trial (1.17 ± 0.05) compared to the first one (1.46 ± 0.059) (*p* = 0.01). In addition, the *post hoc* analysis of CONDITION revealed that participants scaled their load forces significantly better for ERROR (1.05 ± 0.06) than for SOLO (1.58 ± 0.15) (*p* = 0.01). Both ERROR and SOLO did not differ significantly from SKILLED (1.31 ± 0.11) (both *p* > 0.34) (peak LFr values are shown on Figure 3A).

#### Grip Force Rates

Both main effects, as well as the double interaction effect, were significant; CONDITION (*F*_(2,24)_ = 12.81, *p* < 0.001, η^2^*_*p*_* = 0.52), REPETITION (*F*_(1,12)_ = 15.18, *p* < 0.01, η^2^*_*p*_* = 0.56), CONDITION X REPETITION (*F*_(2,24)_ = 4.10, *p* = 0.03, η^2^*_*p*_* = 0.25). Significant findings are discussed in light of the double interaction effect. As can be seen in Figure 3B, it is noticeable that participants scaled their grip forces significantly better after observing an erroneous lift (first ERROR trial), in comparison with both SKILLED and SOLO condition (first SKILLED and SOLO trials) (both *p* < 0.05). Moreover, participants improved from their first to second trial in both SKILLED and SOLO (both *p* < 0.02) but not in the ERROR condition (*p* = 1). Importantly, the *post hoc* analysis did not reveal any significant differences between the second SOLO trial and the first SKILLED and ERROR trial (first SOLO vs. first SKILLED and ERROR) (both *p* = 1) (Table 1). Accordingly, these findings indicate that observing either performance type (first SKILLED or ERROR trial) mediated similar information as tactile feedback (second SOLO trial which participants performed after having had tactile feedback from the first SOLO trial).

#### Loading Phase Duration

Again, all effects were significant; CONDITION (*F*_(2,24)_ = 6.94, *p* < 0.001, η^2^*_*p*_* = 0.37), REPETITION (*F*_(1,12)_ = 54.04, *p* < 0.001, η^2^*_*p*_* = 0.82), CONDITION X REPETITION (*F*_(2,24)_ = 12.00, *p* < 0.001, η^2^*_*p*_* = 0.50). In line with the findings for grip force rates, LPD was significantly longer after observing an erroneous lift (first ERROR trial), compared to having no observation (first SOLO trial) or after having observed a skilled lift (first SKILLED trial) (*p* < 0.01) (Figure 3C). This indicates that participants scaled their forces more for the light than for the heavy weight. These findings are further substantiated as the *post hoc* analysis revealed that participants significantly improved in SKILLED and SOLO (first SKILLED and SOLO trial vs. second one of respective condition) (*p* < 0.001), which did not happen in ERROR (*p* = 1.00). Moreover, the analysis also revealed that the first ERROR trial did not differ significantly from the second SOLO trial (*p* = 1.00) but also that the first SKILLED and second SOLO trial differed significantly (*p* < 0.01), indicating that only observing errors (first ERROR trial) mediate LPD similarly, as having tactile feedback (second SOLO trial). Lastly, it is noteworthy that participants overcompensated in the second trial after the weight change, as shown by the significant difference between the second SKILLED and SOLO trials (*p* < 0.01).

In conclusion, these results indicate that observing erroneous lifts but not skilled lifts improve predictive object lifting. Moreover, our results suggest that observing skilled lifts might improve predictive grip force scaling in the observer.

### Observers’ Lifting Force Parameters: Heavy-After-Light Weight Changes

The right panels of Figure 2 show the averaged force profiles of the first trials of each condition after the object weight changed from light to heavy in a typical participant. When a participant scales his fingertip forces in anticipation of a light object (although it is actually heavy) less force than required (undershoot) will be applied to lift the heavy object (Johansson and Westling, 1988). Figure 2 suggests that the participant upscaled his force generation after observing an erroneous or skilled lift compared to the SOLO condition. Please note that the second trial is not shown on Figure 2; but improvements from trial 1 to trial 2 for each condition is shown on Supplementary Figure S2. In the case of heavy-after-light-weight changes, force parameters with ratios <1 indicate typical weight underestimation effects. These effects are the opposite for loading phase duration: a ratio value >1 indicates a longer LPD caused by the slower scaling of the fingertip forces. All group averages of the participants for the light-after-heavy weight changes can be found in Table 1.

#### Load Force Rate

**FIGURE 4:**
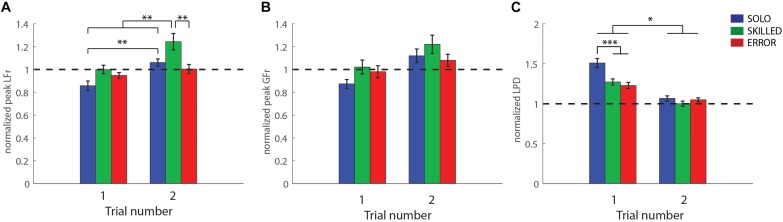
Heavy-after-light weight changes: Participant group averages for the first and second trials after the weight changed from light-to-heavy for the three conditions [grasping with incorrect weight expectations (SOLO), grasping after observing a skilled lift (SKILLED) and grasping after observing a lifting error (ERROR)]. **(A)** Peak load force rates, **(B)** peak grip force rates and **(C)** loading phase durations. All data is represented as a ratio (normalized to skilled lifting; first and second trial of the participants divided by the third trial of the same weight sequence block). A ratio <1 for peak grip force rates and peak load force rates (and a ratio >1 for loading phase durations) indicates that participants underestimated object weight. All data is presented as the pooled mean ± SEM. ^∗∗∗^*p* < 0.001, ^∗∗^*p* < 0.01, ^∗^*p* < 0.05.

Analysis of peak load force rate revealed that both main and the interaction effect were significant, CONDITION (*F*_(2,24)_ = 6.48, *p* < 0.01, η^2^*_*p*_* = 0.35), REPETITION (*F*_(1,12)_ = 20.61, *p* < 0.001, η^2^*_*p*_* = 0.63), CONDITION X REPETITION (*F*_(2,24)_ = 3.63, *p* = 0.04, η^2^*_*p*_* = 0.23). Our findings are interpreted in light of the double interaction effect. As can be seen in Figure 4A, the *post hoc* analysis indicates participants performed similarly, in the first trial of each condition (all *p* = 1.00). In addition, participants improved from their first to second SOLO trial (*p* = 0.001), from their first to second SKILLED trial (*p* = 0.001), but not from their first to second ERROR trial (*p* = 1.00) (Table 1). However, it is important to note that the improvement in the SKILLED condition is likely caused by participants strongly overcompensating in their second SKILLED trial (value much larger than 1; Table 1). Moreover, both the first SKILLED and ERROR trials had a normalized value close to 1 and did not differ significantly from the second SOLO trial (*p* = 1.00). Accordingly, these results indicate that observing either lifting performance type (first SKILLED and ERROR trials) mediated similar information about object weight as tactile feedback (second SOLO trial).

#### Grip Force Rate

Significance was only found for REPETITION (*F*_(1,12)_ = 25.41, *p* < 0.001, η^2^*_*p*_* = 0.68). Accordingly, these results indicate that participants exerted significantly more force in the second trial (1.14 ± 0.04), irrespective of condition, compared to the first trial (0.96 ± 0.03) (*p* < 0.001) (Figure 4B). However, considering that the normalized value in the first trial is closer to 1, it is unlikely that participants performed “better” in the second trial but rather overcompensated.

#### Loading Phase Duration

In line with the findings for load force rate, significance was found for all effects; CONDITION (*F*_(2,24)_ = 7.19, *p* < 0.01, η^2^*_*p*_* = 0.37), REPETITION (*F*_(1,12)_ = 127.92, *p* < 0.001, η^2^*_*p*_* = 0.91), CONDITION × REPETITION (*F*_(2,24)_ = 8.69, *p* = 0.01, η^2^*_*p*_* = 0.42). As shown in Figure 4C, *post hoc* analysis of the double interaction revealed that participants lifted significantly faster (lower LPD ratio value) after observing either lifting performance type (first SKILLED and ERROR trials), compared to having no observation (first SOLO trial) (both *p* < 0.001). However, for both observation conditions participants improved from the first to the second trial (both *p* < 0.05). In addition, participants lifted significantly slower in their first SKILLED and ERROR trials compared to the second SOLO trial (both *p* < 0.05). As such, these results indicate that lift observation improved lifting performance (first ERROR and SKILLED trials vs. first SOLO trial) but was still inferior to tactile feedback (first ERROR and SKILLED trials vs. second SOLO trial).

In conclusion, our results demonstrated that observing either lifting performance type (error or skilled) can mediate predictive force scaling in the observer for the heavy-after-light-weight change.

### Actor’s Lifting Force Parameters: Light-After-Heavy Weight Changes

The actor only lifted the objects during action observation trials (SKILLED and ERROR conditions). Considering that the actor only made an intentional lifting error in the first trial of ERROR, we expected that this trial would differ significantly from all other trials of both ERROR and SKILLED conditions. Accordingly, all trials, except the first one of ERROR, should not differ significantly from each other. Considering that we normalized our data, the force parameters (peak GFr and LFr) of the first ERROR trial should have a value >1, whereas the LPD value should be <1. All other trials should have values close to 1. Please note that all mean ± SEM values for the actor can be found in Table 2. Lastly, as mentioned in the methods, we included 3 trials for REPETITION (first, second and last trial after the weight change). The last trial was included to check the consistency of the actor throughout a weight sequence. Again, please note that the first and second actor trials actually refer to the first and third trial after the weight change (considering that the participants performed trials 2 and 4).

**TABLE 2:** The actor’s lifting performance, pooled across participants, is presented as mean ± SEM.

		**Skilled lift observation**			**Erroneous lift observation**		
			
		**Trial 1**	**Trial 2**	**Last trial**	**Trial 1**	**Trial 2**	**Last trial**
Light after heavy	pLFr	1.05 ± 0.07	1.18 ± 0.06	1.06 ± 0.03	11.96 ± 0.91^∗^	1.05 ± 0.05	1.01 ± 0.04
	pGFr	1.08 ± 0.07	1.14 ± 0.05	0.98 ± 0.04	5.46 ± 0.34^∗^	1.21 ± 0.06	1.18 ± 0.11
	LPD	1.19 ± 0.04	1.06 ± 0.04	1.01 ± 0.02	0.24 ± 0.04^∗^	0.99 ± 0.3	1.05 ± 0.02
Heavy after light	pLFr	1.11 ± 0.05	1.14 ± 0.05	1.01 ± 0.02	1.02 ± 0.06	1.23 ± 0.06	1.00 ± 0.03
	pGFr	1.26 ± 0.06	1.16 ± 0.05	1.05 ± 0.04	0.81 ± 0.04^∗^	1.26 ± 0.06	1.06 ± 0.05
	LPD	0.97 ± 0.04	0.94 ± 0.02	1.03 ± 0.01	1.97 ± 0.07^∗^	0.86 ± 0.02	1.05 ± 0.02

*Values represent the normalized peak load force rate (pLFr), peak grip force rate (pGFr) and loading phase duration (LPD) for the first, second and last trials of the weight sequence blocks of both dyadic conditions (SKILLED: skilled lift observation, ERROR: erroneous lift observation). Normalization was done against the third trial after the weight change. Values above 1 for the peak force values indicate that the actor used more force than required, which should be opposite for LPD (lower than 1). In contrast, values below 1 for the peak force values indicate that the actor used less force required (above 1 for LPD). As the actor only made lifting errors during the first trial of the ERROR condition, this trial should significantly differ from the parameters of all other trials of both the SKILLED and ERROR condition. ^∗^Indicates whether the first trial of the ERROR condition differs significantly from the same parameters of all other trials.*

#### Load Force Rates

**FIGURE 5:**
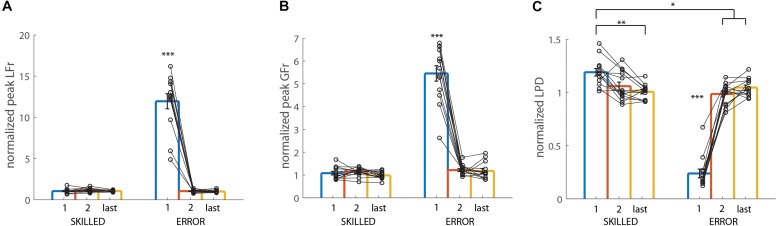
Actor data: Light-after-heavy weight changes: Averaged lift performance for the actor pooled across all participants for light object lifts. **(A–C)** Show averaged data for peak LF rate (LFr), peak GF rate (GFr), and LPD, respectively. All data is represented as a ratio (normalized to skilled lifting; first second and last trial divided by the third trial of the same weight sequence block). Left bars (SKILLED) represent lifts performed by the actor in the skilled condition, right bars (ERROR), represent lifts performed by the actor in the error condition. All data is presented as the pooled mean ± SEM. Two connected scatter plots (one for SKILLED and one for ERROR) indicate the actor’s performance for one participant. As the actor should have only made lifting errors in the first trial of ERROR, only this trial should differ significantly from all others. ^∗∗∗^*p* < 0.001, ^∗∗^*p* < 0.01, ^∗^*p* < 0.05. When the asterisk is placed above one bar only, this indicates that this trial significantly differed from all others.

The repeated-measures ANOVA revealed that all effects, i.e., CONDITION (*F*_(1,12)_ = 124.05, *p* < 0.001, η^2^*_*p*_* = 0.91), REPETITION (*F*_(2,24)_ = 138.20, *p* < 0.001, η^2^*_*p*_* = 0.92) and CONDITION × REPETITION (*F*_(2,24)_ = 149.30, *p* < 0.001, η^2^*_*p*_* = 0.93) were significant. In order to compare differences between the different trials of SKILLED and ERROR, we primarily investigated the *post hoc* analysis of the double interaction effect. As shown in Figure 5A, the actor’s peak LFr was significantly higher in the first trial of ERROR compared to all other trials of both conditions (*all p* < 0.001). In addition, no other significant differences were found (all *p* = 1).

#### Grip Force Rates

Again, all effects were significant; CONDITION (*F*_(1,12)_ = 112.90, *p* < 0.001, η^2^*_*p*_* = 0.90), REPETITION (*F*_2,24)_ = 176.49, *p* < 0.001, η^2^*_*p*_* = 0.94), CONDITION × REPETITION (*F*_(2,24)_ = 122.52, *p* < 0.001, η^2^*_*p*_* = 0.92). Identical to the findings for peak LFr, the *post hoc* analysis of the double interaction effect shows that the actor scaled his grip forces significantly faster (higher peak GFr value) in the first ERROR trial, compared to all other trials of both conditions (all *p* < 0.001). Again, no other significant differences were found between the other trials (all *p* = 1) (Figure 5B).

#### Loading Phase Duration

Significance was again found for all effects; CONDITION (*F*_(1,12)_ = 78.42, *p* < 0.001, η^2^*_*p*_* = 0.87), REPETITION (*F*_2,24)_ = 128.39, *p* < 0.001, η^2^*_*p*_* = 0.91), CONDITION × REPETITION (*F*_(2,24)_ = 170.55, *p* < 0.001, η^2^*_*p*_* = 0.93). The *post hoc* analysis of the double interaction effect revealed that the actor had a significantly shorter LPD compared to all other trials (all *p* < 0.001). However, the actor lifted the weight significantly slower (higher LPD value) in the first trial of SKILLED, compared to all other trials of both conditions (all *p* < 0.03), except for the second trial of SKILLED (*p* = 0.64) (Figure 5C).

In sum, these results indicate that the actor scaled his fingertip forces (peak GFr and LFr) significantly faster in the first ERROR trial, resulting in a shortened LPD. Importantly, this “overshoot” is considered to be typical for object lifting when the object weight is overestimated. However, our analysis also revealed for LPD that the actor lifted more slowly in the first SKILLED trial compared to all other trials, except for the second SKILLED trial. These findings indicate that the actor might have “overcompensated” to ensure not making the typical lifting error related to the light-after-heavy weight change.

### Actor’s Lifting Force Parameters: Heavy-After-Light Weight Changes

#### Load Force Rates

**FIGURE 6:**
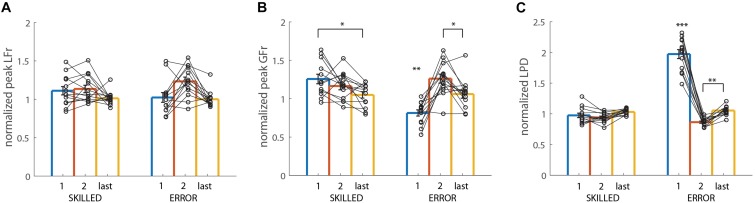
Actor data: Heavy-after-light weight changes: Averaged lift performance for the actor pooled across all participants for heavy object lifts. **(A–C)** Show averaged data for peak LF rate (LFr), peak GF rate (GFr), and LPD, respectively. All data is represented as a ratio (normalized to skilled lifting; first second and last trial divided by the third trial of the same weight sequence block). Left bars (SKILLED) represent lifts performed by the actor in the skilled condition, right bars (ERROR), represent lifts performed by the actor in the error condition. All data is presented as the pooled mean ± SEM. Two connected scatter plots (one for SKILLED and one for ERROR) indicate the actor’s performance for one participant. As the actor should have only made lifting errors in the first trial of ERROR, only this trial should differ significantly from all others. ^∗∗∗^*p* < 0.001, ^∗∗^*p* < 0.01, ^∗^*p* < 0.05. When the asterisk is placed above one bar only, this indicates that this trial significantly differed from all others.

The repeated measures ANOVA only found significance for the main effect of REPETITION (*F*_2,24)_ = 9.49, *p* < 0.001, η^2^*_*p*_* = 0.44), for which the *post hoc* analysis revealed that pLFr was significantly higher for the actor’s second trial (pooled for condition) compared to the first and last trial (both *p* < 0.03). Figure 6A shows the plot of the double interaction effect (which was not significant). As such, no significant difference between the trials of ERROR and SKILLED are discussed.

#### Grip Force Rates

In line with the findings for the light-after-heavy weight changes, all effects were significant; CONDITION (*F*_(1,12)_ = 5.81, *p* = 0.03, η^2^*_*p*_* = 0.33), REPETITION (*F*_2,24)_ = 7.78, *p* < 0.01, η^2^*_*p*_* = 0.39), CONDITION × REPETITION (*F*_(2,24)_ = 29.37, *p* < 0.001, η^2^*_*p*_* = 0.71). *Post hoc* analysis of the double interaction effect shows that peak GFr was significantly smaller in the first ERROR trial compared to all other trials (all *p* < 0.01). However, the actor scaled his grip forces significantly faster (higher pGFr value) in the first trial of the SKILLED compared to the last trial of the same condition (*p* = 0.01). Again, this might indicate that the actor might have subconsciously “overcompensated” in his force scaling to ensure that he would not make the typical “undershooting” error for the heavy-after-light-weight changes. Lastly, the actor scaled his grip forces significantly faster in the second ERROR trial compared to the first and last one of the same condition (both *p* < 0.02). As can be seen on Figure 6B and in Table 2, the actor made a “lifting error” in the first trial of ERROR, then overcompensated by scaling his fingertip forces significantly faster in the second trial, to then return to his “natural” grasping pattern in the last trial (value close to the normalized value of 1).

#### Loading Phase Duration

In line with the findings for grip force rates, all effects were significant; CONDITION (*F*_(1,12)_ = 107.81, *p* < 0.001, η^2^*_*p*_* = 0.90), REPETITION (*F*_2,24)_ = 139.84, *p* < 0.001, η^2^*_*p*_* = 0.92), CONDITION × REPETITION (*F*_(2,24)_ = 180.05, *p* < 0.001, η^2^*_*p*_* = 0.94). The *post hoc* analysis showed that the actor lifted the heavy weight significantly more slowly in the first ERROR trial compared to all other trials of both conditions (all *p* < 0.001). Lastly and similar to the results of peak GFr, the actor overcompensated in the second ERROR trial by lifting significantly faster compared to the last trial of ERROR (*p* < 0.01) (Figure 6C).

All in all, these findings are similar to those for the light-after-heavy weight changes by showing that the actor’s lift performance was different in the first ERROR trial compared to all other trials. Although no significant differences were found for peak LFr, the actor scaled his grip forces significantly slower and also had a slower lifting movement in the first ERROR trial. As such, these results indicate that the actor performed the typical lifting errors consistently for both the heavy-after-light and light-after-heavy-weight changes. However, considering that the lifting errors were intentionally (artificially) performed, we will discuss possible consequences further in the discussion.

## Discussion

The present study investigated whether observation of skilled object lifting allows individuals to update their internal sensorimotor representations, which in turn might translate into changes in anticipatory motor control. Importantly, our results not only corroborate recent findings regarding observation of lifting errors (e.g., Reichelt et al., 2013; Buckingham et al., 2014) but also revealed that observation of natural, skilled hand movements can drive predictive motor control, albeit to a smaller extent than the observation of salient movement errors. For this reason, our results not only support the current consensus that grasp observation allows for accurate weight judgment (e.g., Shim and Carlton, 1997; Meulenbroek et al., 2007) but also sheds new light on the role of more natural movement cues in mediating motor planning in an observer (Uçar and Wenderoth, 2012; Reichelt et al., 2013; Buckingham et al., 2014).

The first aim of our study was to replicate the results of Reichelt et al. (2013). Using a dyadic setting, consisting of a participant and an actor, these researchers revealed that the observation of lifting errors can be used to perceive object weight and, subsequently, allow participants to scale their fingertip forces accurately when lifting the object themselves. When an object with unknown weight was presented, the actor would make a typical lifting error (over- or underestimation of object weight), as he did not have prior knowledge about the object weight (Reichelt et al., 2013). It is plausible that participants deduced object weight based on the observed kinematics: firstly, it has been well established that over- and underestimation of object weight shortens or elongates the lifting phase, respectively, when lifting an object (for example see: Johansson and Westling, 1988; Gordon et al., 1991). Secondly, Hamilton et al. (2007) demonstrated that individuals will estimate an object to be light when they observe a short lifting phase and, conversely, will estimate an object to be heavy when observing a longer lifting phase (Hamilton et al., 2007). Our results are consistent with the findings of Reichelt et al. (2013): Participants in the current study were capable to predictively scale their fingertip forces with significantly improved accuracy after observing a shortened or elongated lifting phase (i.e., lifting errors), indicating a change in object weight. Secondly, we wanted to demonstrate that the observation of skilled lifts can mediate predictive force planning in the observer as well. Importantly, our results indicate that observing a skilled lift of a heavy object (i.e., heavy-after-light change) improves predictive force scaling. We did not find similar results for the light-after-heavy-weight change. However, it is noteworthy that participants scaled their grip forces (peak GFr) significantly better after observing a skilled lift (first SKILLED trial), compared to when they could not expect the weight change (first SOLO trial). As such, our results indicate that observing skilled lifts for the light-after-heavy weight change might still improve predictive grip force planning.

In conclusion, our results show that observation of either lift performance type (erroneous or skilled) can improve predictive object lifting in the observer, similarly to when tactile feedback during an actual lift experience can be used to improve the next lift. However, considering that we only found these results for the heavy-after-light-weight change (but not light-after-heavy), our results indicate that the information provided by skilled lift observation seems to be less impactful in mediating sensorimotor memory compared to observation of erroneous lifts or actual tactile feedback.

It is noteworthy that our results about skilled grasp observation are in contrast with the study of Buckingham et al. (2014). Indeed, their study revealed that error, but not skilled lift observation, significantly reduced the learning that is required to grasp a novel, surprisingly light object (Buckingham et al., 2014). Importantly, there are two major considerations to take into account while comparing the results of the Buckingham study and ours. Firstly, while we used two differently weighted objects with identical appearance, Buckingham et al. (2014) used two objects that were identical in weight but different in size (i.e., “Size-Weight Illusion”). It is likely that this size difference caused a strong initial bias regarding weight expectations toward the objects (for example see: Gordon et al., 1991; Peters et al., 2016). Secondly, in the Buckingham study, participants were not familiarized with the objects and did not receive any information about lifting performance prior to observing object lifting videos. This lack of familiarization and prior information, as well as the presence of a size-weight illusion, might induce a different action observation strategy for extracting information from skilled or erroneous lifting: when lifting skilfully, the kinematics of the lifting phase tend to have a similar duration regardless of object weight (Gordon et al., 1991). According to this, it is likely that participants observing skilled lifts presumed that size and weight were associated, therefore, leading them to focus on size cues rather than on other relevant cues (such as hand contraction or kinematics) that could have indicated that both objects weighed the same. In contrast, the observation of lifting errors might have revealed that the expected relationship between size and weight was not veridical, which likely led participants to not only focus on size but also on the movement kinematics. In our study, participants could only rely on the observed movement kinematics and hand contraction states to assess object weight as we excluded other visuals cues indicating object weight. Interestingly, participants could perceive object weight during both the observation of skilled and erroneous lifting. For observation of errors, it is likely that participants perceived object weight by focusing on the hand contraction, the lifting phase duration and grasp duration (hand-object contact without movement) (Hamilton et al., 2007; Johansson and Flanagan, 2009). Having experienced these typical lifting errors in the SOLO condition, participants were likely to interpret the lifting errors made by the actor and adjust their internal sensorimotor representation accordingly. In contrast, when observing skilled lifting, the kinematic profiles of a heavy or light lift are more similar, compared with lifting errors on the same objects. It is therefore possible that participants did not only rely on the movement kinematics or hand contraction but potentially developed an observational strategy emphasizing different parameters to differentiate between weights, such as the reaching phase (Ansuini et al., 2014) or the intention of the actor (Cavallo et al., 2016; Finisguerra et al., 2016).

It has recently been demonstrated that the action observation-induced increase of corticospinal excitability in the primary motor cortex, termed as “motor resonance,” reflects specific parameters during grasp observation, such as the hand contraction state (Alaerts et al., 2010b) or observed movement kinematics, indicating object weight (Alaerts et al., 2010b; Senot et al., 2011), object shape (Buckingham et al., 2014) and even the intentions of the observed actor (Finisguerra et al., 2016). In the current study, participants were not able to perceive object weight via intrinsic object properties. For this reason, it is plausible that participants had access to information about object weight by mapping onto their own motor repertoire the observed visuomotor cues, such as object kinematics and hand contraction states.

It is noteworthy that a key limitation to the present study is that we used a confederate rather than a naïve actor, which should have had minimal impact for the skilled observation condition. By placing the cubes into the manipulandum, the sensorimotor memory of the actor should always have been correctly updated for the upcoming trial. For the ERROR condition, it is important to note that the actor’s artificial errors are produced in the appropriate direction. For example, for the light-after-heavy weight change, the actor used significantly more force than required for a skilled lift. However, the artificial lifting errors were largely exaggerated. For example, for participants the LPD of an erroneous lift of a light object was approximately 20% shorter compared to a skilled lift. For the actor, this was about 75%. In line with other studies investigating predictive force planning for object lifting, the lifting errors of the actor are indeed largely exaggerated (for instance see: Johansson and Westling, 1988; Gordon et al., 1991; Reichelt et al., 2013; Buckingham et al., 2014).

It is unsure to which extent participants were biased by the artificial movement kinematics of the actor’s erroneous lifts as, for instance, it has been demonstrated that motor resonance is altered when observing deceptive actions (Tidoni et al., 2013). However, it is important to note that in their study, the actor pretended to lift one weight as the other. For instance, when lifting the heavy weight deceptively as a light one, the actor attempted to have the deceptive lift of a heavy object resemble the truthful lift of a light weight as much as possible. In contrast, in the present experiment, the actor did not attempt to have the “deceptive” (erroneous) lifts resemble each other as much as possible but rather made the kinematic differences between them even larger. Moreover, participants were informed on lifting errors and explained that the actor would only make them in his first trial after the weight change in the error observation condition. As such, participants should have understood that the actor’s artificial lifting errors resembled the lifting errors they made themselves in the SOLO condition. Because of these reasons, we argue that the confounding effect of artificial lifting errors should have been minimal. Importantly, this seems to be supported by the similarity between our findings and those of Reichelt et al. (2013). However, it could be interesting to reproduce the findings of the present experiment with a naïve actor in the error condition to eradicate any potential influence of the actor’s intentions. In addition, it might also be interesting to run the same experiment but with observation of either lifting performance types (error vs. skilled) completely randomized, to ensure subjects cannot predict how weight changes would be indicated to them.

In conclusion, participants in the present study were familiarized to two different object weights and generated a sensorimotor repertoire for skilled lifting (by applying accurate forces following consecutive lifts of a same object) and for lifting errors (by over- or underestimating forces after a weight change). After this initial process, participants lifted objects in turns with an actor. In this dyadic setting, the only way individuals could extract information about weight, and in turn plan their subsequent motor command, was by embodying the observed visuomotor cues into their own sensorimotor repertoire. Our results not only support recent findings regarding the effect of observation of explicit movement errors on mediating predictive motor control but also highlight that the observation of skilled movements, consisting of more subtle differences between lifts of different weights, can also drive motor planning. Interestingly, anticipatory force scaling in the first trial following skilled lift observation was not as accurate as following error observation, and still improved in the second trial. This highlights that different action observation mechanisms could contribute to mediating anticipatory motor control in an observer when surprising or erroneous movements are performed (Cretu et al., 2019).

## Data Availability Statement

The datasets generated for this study are available on request to the corresponding authors.

## Ethics Statement

The studies involving human participants were reviewed and approved by Ethical Committee of the Faculty of Biomedical Sciences, KU Leuven. The patients/participants provided their written informed consent to participate in this study.

## Author Contributions

Both authors contributed to the conception and design of the study, revised the manuscript, read and approved the submitted version. GR performed the experiments and statistical analysis and wrote the first draft of the manuscript.

## Conflict of Interest

The authors declare that the research was conducted in the absence of any commercial or financial relationships that could be construed as a potential conflict of interest.

## Supplementary Material

FIGURE S1 Light-after-heavy weight changes: Participants group averages combined with connected scatter plot (each participant represents one color) after the weight changed from heavy-to-light for the three conditions [grasping with incorrect weight expectations (SOLO), grasping after observing a skilled lift (SKILLED) and grasping after observing a lifting error (ERROR)]. **(Left)** Peak load force rates, **(middle)** Peak grip force rates and **(right)** loading phase durations. All data is represented as a ratio (normalized to skilled lifting; first and second trial of the participants divided by the third trial of the same weight sequence block). A ratio >1 for peak grip force rates and peak load force rates (and a ratio <1 for loading phase durations) indicates that object weight was overestimated. All data is presented as the pooled mean ± SEM. Data is grouped for condition, so the improvement in lift performance from trial 1 to 2 can be compared for each condition. Significance is not shown on this figure and we refer the reader to Figure 3 for this.

FIGURE S2 Heavy-after-light weight changes: Participants group averages combined with connected scatter plot (each participant represents one color) after the weight changed from light to heavy for the three conditions [grasping with incorrect weight expectations (SOLO), grasping after observing a skilled lift (SKILLED) and grasping after observing a lifting error (ERROR)]. **(Left)** Peak load force rates, **(middle)** Peak grip force rates and **(right)** loading phase durations. All data is represented as a ratio (normalized to skilled lifting; first and second trial of the participants divided by the third trial of the same weight sequence block). A ratio >1 for peak grip force rates and peak load force rates (and a ratio <1 for loading phase durations) indicates that object weight was underestimated. All data is presented as the pooled mean ± SEM. Data is grouped for condition, so the improvement in lift performance from trial 1 to 2 can be compared for each condition. Significance is not shown on this figure and we refer the reader to Figure 4 for this.
