# Supplementary figures and images for: Observation of Both Skilled and Erroneous Object Lifting Can Improve Predictive Force Scaling in the Observer
Source: Front Hum Neurosci. 2019 Oct 22;13:373. doi: 10.3389/fnhum.2019.00373 (PMC6817912; doi:10.3389/fnhum.2019.00373)

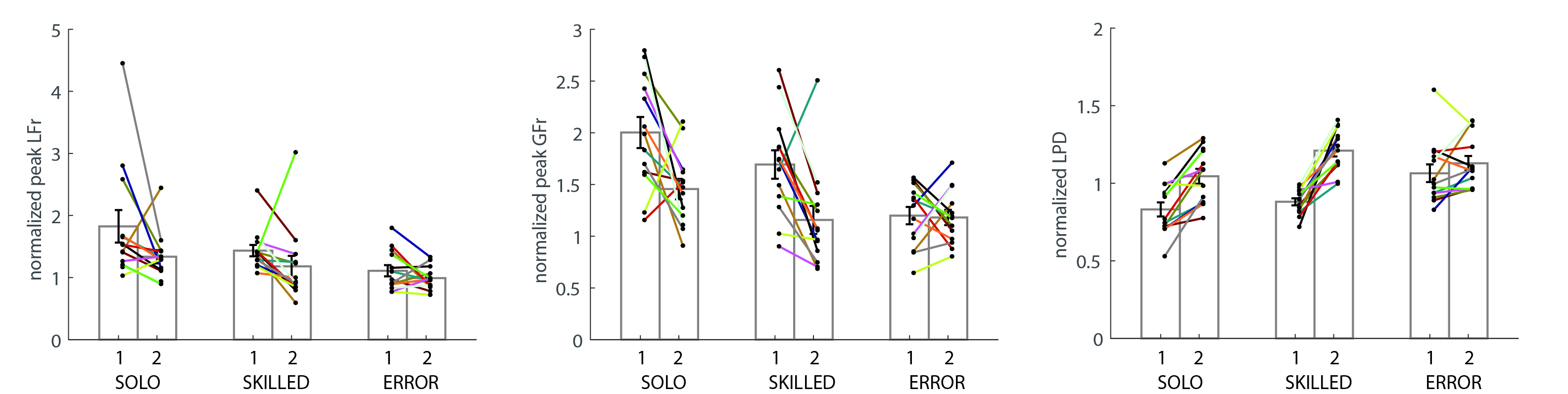

Supplement: FIGURE S1 — Light-after-heavy weight changes: Participants group averages combined with connected scatter plot (each participant represents one color) after the weight changed from heavy-to-light for the three conditions [grasping with incorrect weight expectations (SOLO), grasping after observing a skilled lift (SKILLED) and grasping after observing a lifting error (ERROR)]. (Left) Peak load force rates, (middle) Peak grip force rates and (right) loading phase durations. All data is represented as a ratio (normalized to skilled lifting; first and second trial of the participants divided by the third trial of the same weight sequence block). A ratio >1 for peak grip force rates and peak load force rates (and a ratio <1 for loading phase durations) indicates that object weight was overestimated. All data is presented as the pooled mean ± SEM. Data is grouped for condition, so the improvement in lift performance from trial 1 to 2 can be compared for each condition. Significance is not shown on this figure and we refer the reader to Figure 3 for this. [file Image_1.jpg]

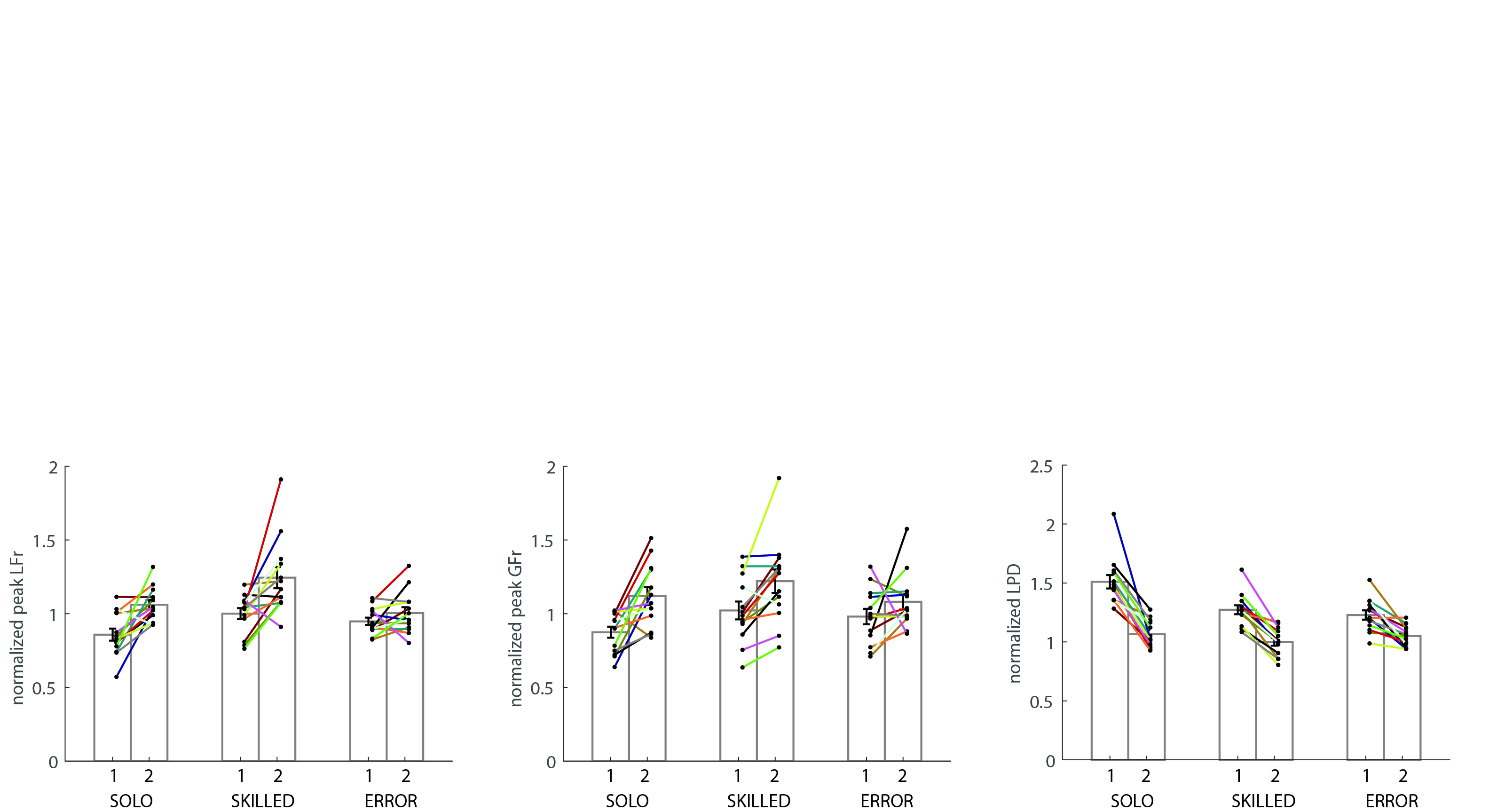

Supplement: FIGURE S2 — Heavy-after-light weight changes: Participants group averages combined with connected scatter plot (each participant represents one color) after the weight changed from light to heavy for the three conditions [grasping with incorrect weight expectations (SOLO), grasping after observing a skilled lift (SKILLED) and grasping after observing a lifting error (ERROR)]. (Left) Peak load force rates, (middle) Peak grip force rates and (right) loading phase durations. All data is represented as a ratio (normalized to skilled lifting; first and second trial of the participants divided by the third trial of the same weight sequence block). A ratio >1 for peak grip force rates and peak load force rates (and a ratio <1 for loading phase durations) indicates that object weight was underestimated. All data is presented as the pooled mean ± SEM. Data is grouped for condition, so the improvement in lift performance from trial 1 to 2 can be compared for each condition. Significance is not shown on this figure and we refer the reader to Figure 4 for this. [file Image_2.jpg]
